# Supplementary figures and images for: Comparison of the Portuguese Version of the Pregnancy Physical Activity Questionnaire (PPAQ) with Accelerometry for Classifying Physical Activity among Pregnant Women with Obesity
Source: Int J Environ Res Public Health. 2023 Jan 4;20(2):929. doi: 10.3390/ijerph20020929 (PMC9859283; doi:10.3390/ijerph20020929)

Supplementary File S1—Bland Altman graphs

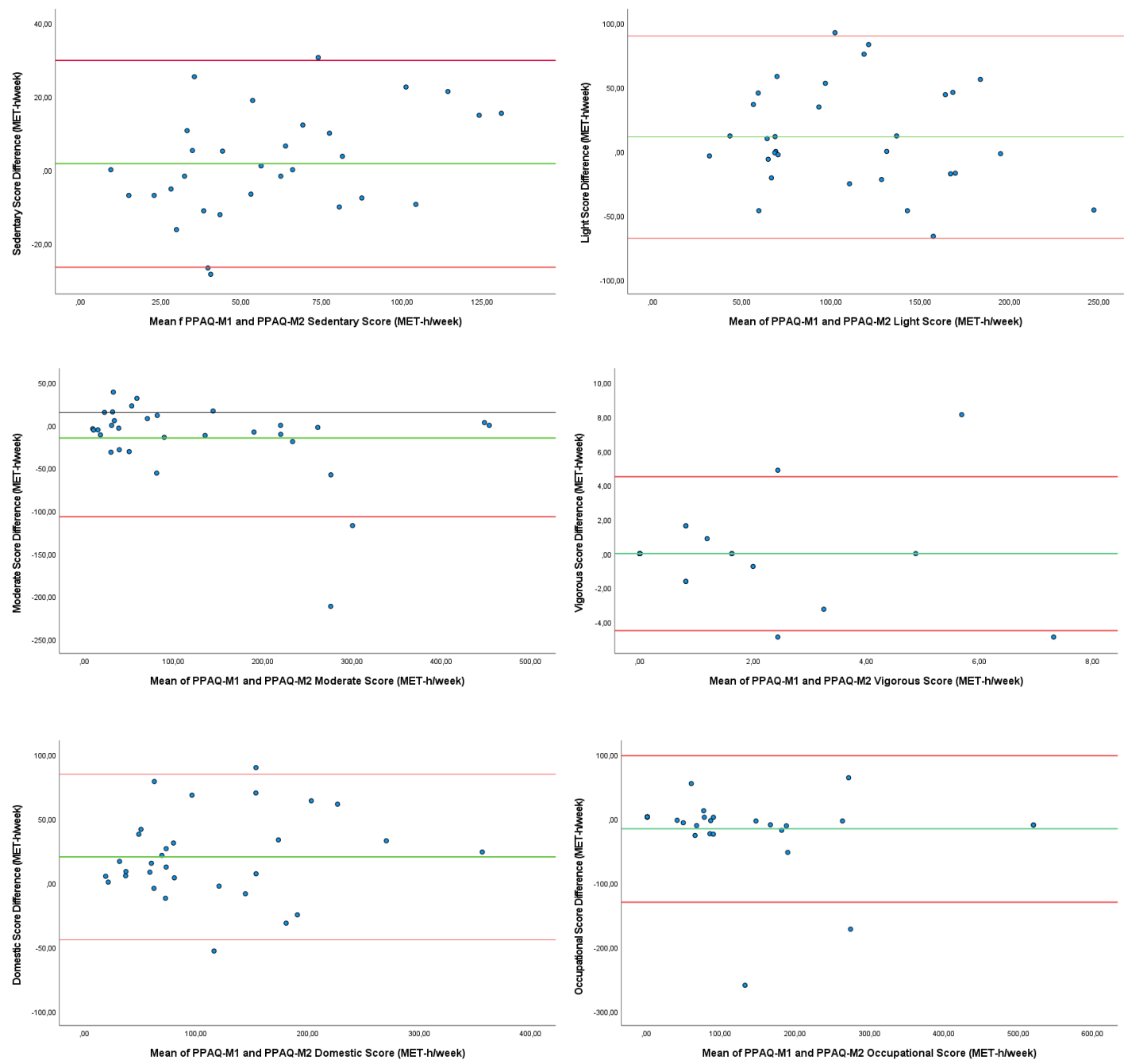

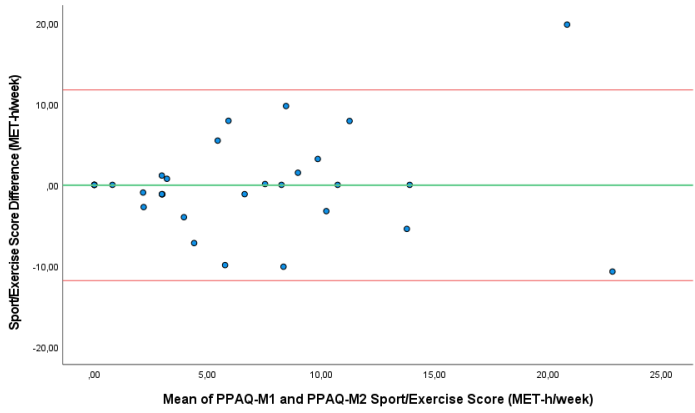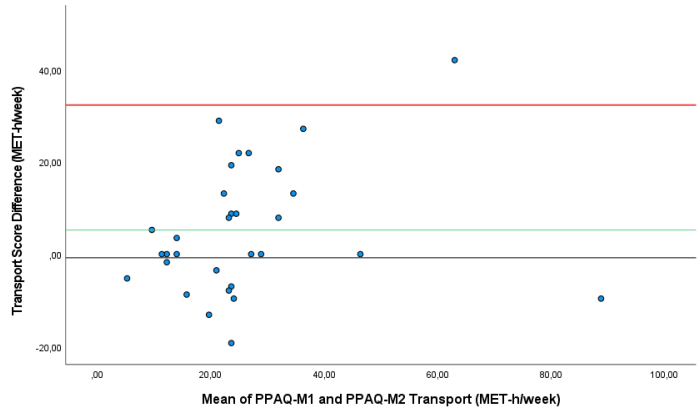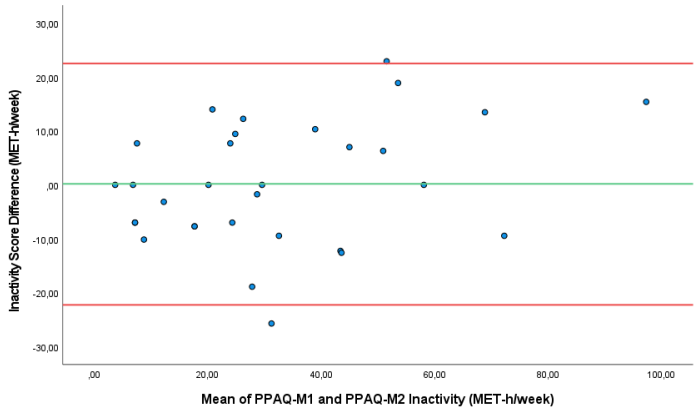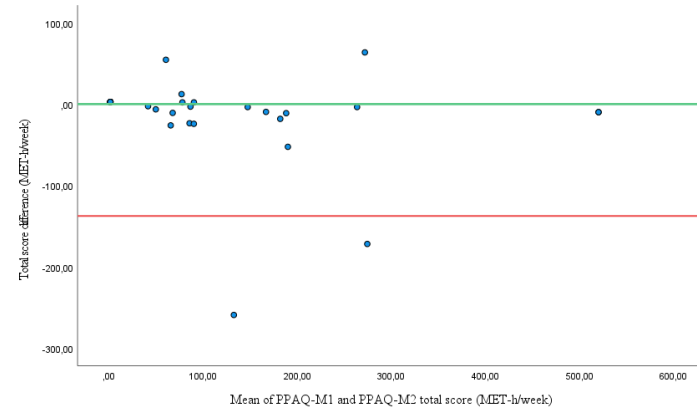

Supplement: Supplementary file 1 [file ijerph-20-00929-s001.zip › ijerph-2058225-supplementary.pdf]
